# The Changing Face of Occupational Medicine

**DOI:** 10.1371/journal.pmed.0040221

**Published:** 2007-06-26

**Authors:** The PLoS Medicine Editors

When most people think about the adult film industry it's likely that few have in mind the rights of workers within this industry. A paper in this month's issue of *PLoS Medicine* [1], however, takes just that point of view and concludes that the time has come for this highly profitable industry to self-regulate, and specifically to address the issue of the health of its workers. The workers whose health most needs to be protected are not those who operate the cameras or build the sets; it's the actors and actresses who run the biggest risks every day in their working lives. Infection with HIV is perhaps the most obvious and most alarming risk of this profession, but adult film performers also risk infection with other sexually transmitted diseases, including chlamydia and gonorrhea [2]. As the authors conclude, “Unfortunately, the growing popularity of adult film has not translated into safer working conditions for performers. It is unethical for industry executives, legislators, and consumers to continue to enjoy the profits, tax revenues, and gratification of adult film without ensuring the safety of performers” [1].

In the western world probably the first time that workers' health really caught the attention of the wider public was in the early 1800s, when campaigners in the UK brought the issue of child chimney sweeps to public attention. The risks to these children's health became well known—respiratory problems, cancer of the scrotum, and deformed limbs being the most common long term risks, if the children survived the shorter-term risks of death from falling, suffocating, or burning while in the chimney. But despite widespread knowledge of these risks, it still took more than 60 years for the legislation that banned the use of children to be enacted. Enforcing the legislation took even longer, for one simple reason: cost. The high cost of mechanical alternatives to human chimney sweeps compared to children meant that enforcement was not really effective until the 1860s, when the imposition of heavy fines made the cost of noncompliance higher than that of compliance.

One driving force behind occupational medicine, as shown by the example of the chimney sweeps, has been the idea of social justice. Its opposing force, the short-term cost to employers of protecting workers, is a recurring factor today. International bodies (such as the International Labour Organisation [ILO, http://www.ilo.org/], an agency of the United Nations) and national bodies (such as the Occupational Safety and Health Administration [OSHA, http://www.osha.gov/] in the US, and the Health and Safety Executive [http://www.hse.gov.uk/] in the UK) have a relatively recent history and rather checkered success, even in the developed world, at persuading governments and employers that protecting the work force makes long-term financial sense. But work-related accidents are a major cause of death and disability. According to the ILO, every year more than 2 million people die of work-related accidents and diseases and more than 160 million workers become ill because of workplace hazards [3].

In many countries legislation to protect workers is nonexistent or not enforced; in particular, the 1.7 billion workers of the developing world have little or no protection. The most disadvantaged workers of all are migrant workers, who cross the globe in search of work—especially those without legal status. A recent article published as part of a special issue of *PLoS Medicine* on social medicine (http://collections.plos.org/plosmedicine/socialmedicine-2006.php.) documented in migrant Hispanic workers the poor working conditions, poor health care, and discriminatory attitudes that are prevalent in the western US [4]. Europe, too, has many migrant workers and, as so often happens, it took a tragedy to bring these workers' conditions to public attention and to provoke governmental action. The drowning deaths of 23 Chinese cockle pickers in the UK in 2004 led to the introduction of legislation by the UK Government to regulate the conditions in which such workers, under the direction of “gangmasters,” could live and work (see Gangmasters Licensing Authority [http://www.gla.gov.uk/. ]). It is not yet clear how well this legislation is working; a recent journalistic investigation into the conditions of low-paid Lithuanian workers in East Anglia found unsafe working and living conditions [5].

Occupational health is not a minority interest but one that must involve all of society. Nationally and globally, the health of wider populations is inseparable from that of workers. Two further examples, one from the developing and one from the developed world, illustrate this inevitable link. The first example is that of traffic injury, discussed by another paper in this issue that documents the increasing burden of traffic injury in Africa [6]. As an increasingly transitory work force of illegal and unprotected workers takes to the road to seek work, it will disproportionately bear the burden of such injury, as poignantly exemplified in a recent news report in The Bangkok Post [7]:

*“Four immigrant workers were killed yesterday when a pick-up truck they were travelling in crashed and overturned … The bodies of the victims were scattered on the road and the pick-up truck, without a licence plate, was discovered upside down near a swamp. Another pick-up truck was found nearby. Police believe the two trucks were carrying at least 20 illegal migrant workers when the accident took place.”*

In one part of the developed world, workers' health has climbed up the political agenda because of the wider public health interest in the politically charged issue of smoking. In July 2007, England will become the last of the countries of the UK to enforce a smoking ban in enclosed public places. This is a great piece of public health legislation, but it is also a great piece of occupational health legislation, as undoubtedly those who will benefit most are workers in the hospitality industry. It was notable that when the Act was being debated publicly, professional organizations representing occupational health physicians lobbied successfully to have almost complete coverage of the ban despite an initial intention in the Act to exclude some work places [8]. This is occupational medicine at its best and points the way to the future of the profession.

The labor market is becoming more complex and, as highlighted by the adult film industry, is throwing up new and unexpected challenges that health professionals and society at large need to be able to respond to. Most importantly, occupational medicine cannot be a luxury for the developed world or for only certain groups of workers: the right to work in safety is a basic human right, and protection of the health of workers is ultimately of benefit to all of society.
